# HIV/AIDS in Vancouver, British Columbia: a growing epidemic

**DOI:** 10.1186/1477-7517-6-5

**Published:** 2009-03-05

**Authors:** Colin W McInnes, Eric Druyts, Stephanie S Harvard, Mark Gilbert, Mark W Tyndall, Viviane D Lima, Evan Wood, Julio SG Montaner, Robert S Hogg

**Affiliations:** 1 British Columbia Centre for Excellence in HIV/AIDS, St. Paul's Hospital, 608-1081 Burrard Street, Vancouver, British Columbia, V6Z 1Y6, Canada; 2 Faculty of Medicine, University of British Columbia, 3300-950 West 10th Avenue, Vancouver, British Columbia, V5Z 4E3, Canada; 3 Division of STI/HIV Prevention and Control, British Columbia Centre for Disease Control, 655 West 12th Avenue, Vancouver, British Columbia, V5Z 4R4, Canada; 4 Faculty of Health Sciences, Simon Fraser University, 8888 University Drive, Burnaby, British Columbia, V5A 1S6, Canada

## Abstract

The prevalence of HIV in Vancouver, British Columbia was subject to two distinct periods of rapid increase. The first occurred in the 1980s due to high incidence among men who have sex with men (MSM), and the second occurred in the 1990s due to high incidence among injection drug users (IDU). The purpose of this study was to estimate and model the trends in HIV prevalence in Vancouver from 1980 to 2006. HIV prevalence data were entered into the UNAIDS/WHO Estimation and Projection Package (EPP) where prevalence trends were estimated by fitting an epidemiological model to the data. Epidemic curves were fit for IDU, MSM, street-based female sex trade workers (FSW), and the general population. Using EPP, these curves were then aggregated to produce a model of Vancouver's overall HIV prevalence. Of the 505 000 people over the age of 15 that reside in Vancouver, 6108 (ranging from 4979 to 7237) were living with HIV in the year 2006, giving an overall prevalence of 1.21 percent (ranging from 0.99 to 1.43 percent). The subgroups of IDU and MSM account for the greatest proportion of HIV infections. Our model estimates that the prevalence of HIV in Vancouver is greater than one percent, roughly 6 times higher than Canada's national prevalence. These results suggest that HIV infection is having a relatively large impact in Vancouver and that evidence-based prevention and harm reduction strategies should be expanded.

## Background

In Vancouver, British Columbia, the population subgroups most affected by HIV have experienced different rates of infection over the course of the epidemic. In the 1980s, most HIV infections were accounted for by sexual transmission among men who have sex with men (MSM), and in the mid-1990s a rapid increase in HIV incidence was observed among injection drug users (IDU) and street-based female sex trade workers (FSW) [1,2]. Although this shift in HIV trends was well documented, it has not been adequately quantified or characterized in the historical context of the city's HIV epidemic.

Measuring longitudinal trends in the prevalence of HIV is essential to characterize the epidemic and to monitor changes in high-risk population subgroups. As disease prevalence reflects both incidence and mortality rates, monitoring trends in HIV prevalence can provide insight into the impact of events affecting HIV risk as well as survival, such as increased use of injection cocaine or the introduction of highly active antiretroviral therapy (HAART). Documenting HIV prevalence over time also provides the denominator needed to calculate HIV-related health indicators within a temporal frame, such as the proportion of infected individuals receiving HAART or the proportion with co-infections.

Despite the importance of measuring overall HIV prevalence, current prevalence data in Vancouver have been limited to specific population subgroups and specific points in time. The purpose of this study was to combine estimates of HIV prevalence among population subgroups in Vancouver in order to model the prevalence trends of these subgroups and Vancouver's overall population longitudinally from 1980 to 2006.

## Methods

A literature search was conducted using medical and scientific databases (PubMed, Web of Science), national websites (Public Health Agency of Canada, Statistics Canada), and a general search engine (Google) in order to identify all published and unpublished estimates of HIV prevalence in Vancouver among MSM, IDU, FSW, and pregnant women receiving antenatal testing (PW), a lower risk population subgroup and reference point for the remaining general population. Published and unpublished estimates of MSM, IDU, and FSW population sizes were also extracted. All data sources of HIV prevalence and population sizes are listed in Table 1. Since the HIV prevalence among PW was used to reflect the prevalence of the lower risk, remaining population, they were assigned a large population size. Population estimates for the city of Vancouver were taken from Statistics Canada [3]. The final population subgroup sizes were based on previously published estimates and peer-based discussions. Individuals under 15 years of age were not included in our analyses as data on this group are limited.

**Table 1:** Model parameters and data sources of subgroup population sizes and HIV prevalence data

Key parameters	Sources
Vancouver	
*population size*	Statistics Canada [3]
	
MSM*	
*population size*	Population surveys, capture-recapture estimates [11,19]
*HIV prevalence*	Cohort and cross-sectional surveys [20-23]
	
IDU*	
*population size*	Population surveys, capture-recapture estimates [11,24]
*HIV prevalence*	Cohort and cross-sectional surveys [1,25-29]
	
FSW*	
*population size*	Peer-based discussions
*HIV prevalence*	Community-based studies of FSW [1,25,30]
	
General population*†	
*population size*	Remaining population
*HIV prevalence*	Antenatal seroprevalence studies [31,32]

*HIV prevalence estimates each included data from the Health Canada Inventory of HIV Prevalence Studies [33].

†The prevalence of pregnant women receiving antenatal testing was used to impute the estimate for the general population.

MSM, men who have sex with men; IDU, injection drug users; FSW, street-based female sex trade worker.

The HIV prevalence input assumptions for the year 2006 were based on the most recent available measures, all of which were from the year 2003 or later. Vancouver's total population over the age of 15 was estimated to be 505 000 [3]. The estimated sizes of the population subgroups are as follows: MSM 20 000 (15 000 – 25 000), IDU 13 500 (10 000 – 15 000), FSW 1500 (1000 – 2000), PW 470 000 (477 000 – 463 000). All estimates of HIV and population sizes are presented in Table 2.

**Table 2:** Estimated population subgroup sizes and HIV prevalence for persons 15 years of age and older living in Vancouver

**Transmission group**	**Estimated population size (low and high estimates)**	HIV prevalence, 2006*
MSM	20 000 (15 000 – 25 000)	15.0%
IDU	13 500 (12 000 – 15 000)	17.0%
FSW	1500 (1000 – 2000)	26.0%
General population†	470 000 (477 000 – 463 000)	0.09%

*Prevalence input assumptions for the year 2006 were based on the most recent available measures, all of which were from the year 2003 or later.

†The prevalence of pregnant women receiving antenatal testing was used to impute the estimate for the general population.

MSM, men who have sex with men; IDU, injection drug users; FSW, street-based female sex trade worker.

All data were entered into the UNAIDS/WHO Estimation and Projection Package (EPP) [4], where prevalence trends for each population subgroup were estimated longitudinally by fitting an epidemic curve to the data for each subgroup. EPP finds the curve of best fit by minimizing the log likelihood of several parameters, such as the start year of the epidemic and the rate of HIV transmission. The epidemic curves that were modeled for population subgroups were aggregated by EPP to find the best fitting curve that models the overall trends of Vancouver's HIV prevalence. Based on the estimated population sizes (Table 2), a low growth model, a high growth model, and an intermediate model, reflecting our best estimate, were produced for the overall population.

## Findings

Table 3 provides the estimates of the number of HIV-infected individuals from the specific population subgroups and Vancouver's entire population for the year 2006. We estimate that a total of 6108 (ranging from 4979 to 7237) men and women were living with HIV in the year 2006, producing an overall HIV prevalence of 1.21% (ranging from 0.99% to 1.43%). Our models estimate that MSM and IDU subgroups contributed the greatest number of infections, with 3000 (ranging from 2250 to 3750) and 2295 (ranging from 2040 to 2550) individuals, respectively.

**Table 3:** Estimated number of persons infected with HIV in Vancouver, 2006

**Variable**	**HIV Infected**		
	
	**Low Estimate**	**Middle Estimate**	**High Estimate**
*Transmission groups*			
MSM	2250	3000	3750
IDU	2040	2295	2550
FSW	260	390	520
General population†	429	423	417
			
*Total population*			
Males	3585	4459	5355
Females	1394	1649	1882
Total infected	4979	6108	7237

Overall prevalence	0.99%	1.21%	1.43%

†The prevalence of pregnant women receiving antenatal testing was used to impute the estimate for the general population.

MSM, men who have sex with men; IDU, injection drug users; FSW, street-based female sex trade worker.

**Figure 1:**
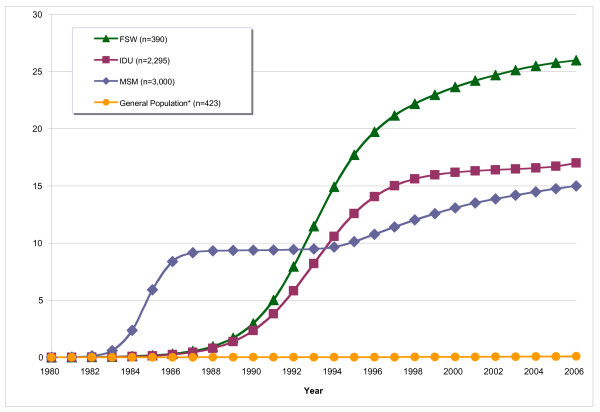
**Middle estimates of HIV prevalence for population subgroups in Vancouver, 1980–2006**. *The prevalence of pregnant women receiving antenatal testing was used to impute the estimate for the general population. MSM, men who have sex with men; IDU, injection drug users; FSW, street-based female sex trade worker; PW, pregnant women receiving antenatal testing.

The EPP model depicting the prevalence of HIV from 1980 to 2006 in each subgroup is shown in Figure 1. The model illustrates the rise in HIV prevalence among MSM in the 1980s as well as the rapid increase in prevalence among IDU and FSW in the 1990s. The upward trends of the model project the potential for moderate increases in HIV prevalence within each of these population subgroups.

**Figure 2:**
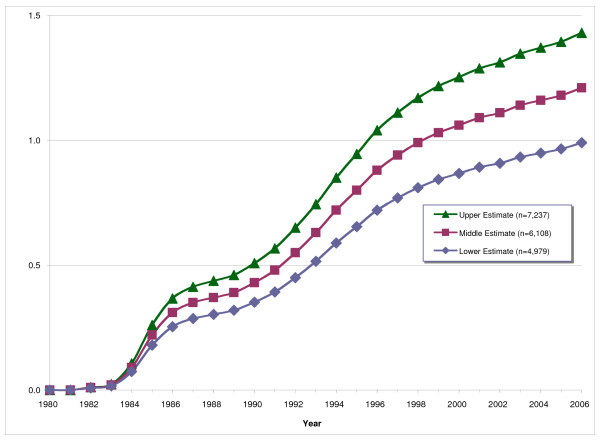
**HIV prevalence among people living in Vancouver by upper, middle and lower estimates, 1980–2006**.

Figure 2 characterizes the trend in Vancouver's overall HIV prevalence since 1980. The model depicts two rapid increases in HIV prevalence, the first in the mid-1980s and the second in the mid-1990s, and the upward trend of the model projects the potential for a moderate future increase in Vancouver's overall HIV prevalence.

## Discussion

In the year 2006, there were approximately 6108 (ranging from 4979 to 7237) people living in Vancouver that were infected with HIV. Populations of IDU and MSM contributed the largest number of infections. Although HIV prevalence was highest among FSW, due to the relatively small size of this subgroup, they accounted for only a small proportion of the total HIV infections in Vancouver. Few total infections were from PW, our reference point for the general population. Our overall estimate indicates that the prevalence of HIV in Vancouver was approximately 1.21% (ranging from 0.99% to 1.43%) in the year 2006 and the upward trend in our model suggests that there is potential for this value to increase slightly in the future.

Our model successfully represents Vancouver's two documented periods of rapid increase in HIV prevalence [1,2]. The first rise in Vancouver's prevalence occurred in the mid-1980s as a consequence of high HIV incidence among MSM [2]. However, the pace of this increase was slowed in the late 1980s, largely as a result of mortality associated with the disease in the MSM subgroup [5]. The second period of rapid increase resulted from high rates of HIV transmission among Vancouver's IDU and FSW subgroups during the mid-1990s, at which time the prevalence of HIV in Vancouver approached 1%. Since this time, our model suggests that the prevalence of HIV in Vancouver has steadily increased and has the potential to increase in the future. Recent increases in prevalence may be attributed to increases in transmission among individuals with detectable HIV viral loads and increased survival among individuals on HAART [6-9].

Vancouver's HIV epidemic remains concentrated in high-risk subgroups of MSM, IDU, and FSW, like other large Canadian cities such as Montreal and Toronto [10]. What distinguishes Vancouver's situation from these other cities, however, is its relatively large population of high-risk individuals, particularly IDU. For example, a previous study [11] estimated that the city of Vancouver has a greater number of IDU than Montreal and a comparable number to that found in Toronto, despite Vancouver having a smaller overall population size. Furthermore, Vancouver's estimated IDU population of 13 500 individuals represents between 11% and 18% of Canada's total IDU population, which has been estimated to be between 75 000 and 125 000 individuals [12]. Given the relatively large IDU and MSM populations in Vancouver, it is not surprising that our model indicates Vancouver's overall HIV prevalence passed the 1% mark in the 1990s, during which time rapid transmission of HIV was observed among IDU [1]. Unfortunately, despite the expansion of needle exchange programs and the implementation of Vancouver's safe injection site, which have both shown the potential to decrease HIV incidence [13-16], transmission of HIV remains high within IDU populations [6], a finding that is reflected in our model of IDU. Similarly, our model suggests that since the mid-1990s the prevalence of HIV among MSM has been increasing, a finding that is also consistent with recent incidence data [7].

As with any estimation of HIV prevalence, the validity of our model is dependent on the validity of the data sources. Unfortunately, due to limitations in the availability of prevalence data, it was necessary to combine different types of data, which may have led to either overestimates or underestimates of prevalence for any of the subgroups. For example, the most recent prevalence data on MSM was derived from self-report of HIV status and these data may underestimate prevalence. Furthermore, because the HIV prevalence among high-risk subgroups is not measured annually, our 2006 input assumptions were based on the most recent available measures, some of which were from the years 2003–2005. It is possible that these values have changed, and there is a need for updated seroprevalence data. Another limitation includes our inability to model the prevalence trends of all high-risk population subgroups, such as individuals with mental health disorders, although this subgroup could potentially overlap with the IDU subgroup. Consequently, the prevalence estimate for Vancouver's total population, which relied on data from pregnant women to reflect HIV prevalence outside of the high-risk population subgroups, may be an underestimate. Finally, our model relied on the UNAIDS EPP program, which is unable to account for all the epidemiological factors that could potentially affect HIV prevalence. Importantly, however, this program provides an accessible method for modeling HIV prevalence and may be useful in other settings.

Our model indicates that the prevalence of HIV in the city of Vancouver is approximately 6 times higher than Canada's national prevalence [17,18]. Further, the upward trend of our model suggests that there is potential for future increases in Vancouver's overall HIV prevalence [19-33]. These findings suggest that HIV infection is having a large impact in Vancouver and that evidence-based prevention and harm reduction strategies, particularly those targeted at high-risk population subgroups, should continue to be expanded and evaluated.

## Competing interests

The authors declare that they have no competing interests.

## Authors' contributions

CWM, ED, SSH, VDL, JSGM, RSH initiated the study. CWM, ED, VDL performed the analyses. CWM, ED, SSH prepared the first draft. MG, MWT, EW, JSGM, RSH reviewed the manuscript for important intellectual content. All authors approved the final manuscript for publication.
